# Supplementary material for: Modeling glioblastoma heterogeneity as a dynamic network of cell states
Source: Mol Syst Biol. 2021 Sep 16;17(9):e10105. doi: 10.15252/msb.202010105 (PMC8444284; doi:10.15252/msb.202010105)
Supplement: Supplementary file 6 — Source Data for Figure 5 [file MSB-17-e10105-s004.zip › Figure5A_sourcedata/GSEA_3017/nosigdb_stateC.GseaPreranked.1622795002785/RICHARDS_NOWAKOWSKI_SCIENCE_2017_MGE_IPC3_UPREG.html]

Details for gene set RICHARDS\_NOWAKOWSKI\_SCIENCE\_2017\_MGE\_IPC3\_UPREG[GSEA]

|  || Dataset | state13017 |
| Phenotype | NoPhenotypeAvailable |
| Upregulated in class | na\_pos |
| GeneSet | RICHARDS\_NOWAKOWSKI\_SCIENCE\_2017\_MGE\_IPC3\_UPREG |
| Enrichment Score (ES) | 0.66050243 |
| Normalized Enrichment Score (NES) | 3.9004571 |
| Nominal p-value | 0.0 |
| FDR q-value | 0.0 |
| FWER p-Value | 0.0 |
Table: GSEA Results Summary

  

Fig 1: Enrichment plot: RICHARDS\_NOWAKOWSKI\_SCIENCE\_2017\_MGE\_IPC3\_UPREG      
 Profile of the Running ES Score & Positions of GeneSet Members on the Rank Ordered List

  

| PROBE | GENE SYMBOL | GENE\_TITLE | RANK IN GENE LIST | RANK METRIC SCORE | RUNNING ES | CORE ENRICHMENT || 1 | RND3 |  |  | 2 | 0.713 | 0.0546 | Yes |
| 2 | HES6 |  |  | 5 | 0.694 | 0.1076 | Yes |
| 3 | CCNE2 |  |  | 15 | 0.570 | 0.1381 | Yes |
| 4 | DLL3 |  |  | 16 | 0.562 | 0.1839 | Yes |
| 5 | CLSPN |  |  | 17 | 0.561 | 0.2296 | Yes |
| 6 | HMGB2 |  |  | 20 | 0.534 | 0.2696 | Yes |
| 7 | CDK6 |  |  | 24 | 0.518 | 0.3064 | Yes |
| 8 | DLX1 |  |  | 32 | 0.469 | 0.3322 | Yes |
| 9 | TMPO |  |  | 49 | 0.432 | 0.3392 | Yes |
| 10 | RAB3IP |  |  | 66 | 0.382 | 0.3420 | Yes |
| 11 | GLCCI1 |  |  | 68 | 0.378 | 0.3710 | Yes |
| 12 | SOX4 |  |  | 80 | 0.362 | 0.3811 | Yes |
| 13 | GINS2 |  |  | 83 | 0.360 | 0.4068 | Yes |
| 14 | STMN1 |  |  | 85 | 0.357 | 0.4342 | Yes |
| 15 | USP1 |  |  | 92 | 0.352 | 0.4522 | Yes |
| 16 | H1FX |  |  | 101 | 0.339 | 0.4658 | Yes |
| 17 | CKB |  |  | 102 | 0.335 | 0.4930 | Yes |
| 18 | INSM1 |  |  | 108 | 0.329 | 0.5110 | Yes |
| 19 | BEST3 |  |  | 110 | 0.327 | 0.5358 | Yes |
| 20 | HAT1 |  |  | 121 | 0.318 | 0.5441 | Yes |
| 21 | FBXW7 |  |  | 122 | 0.316 | 0.5698 | Yes |
| 22 | HMGN2 |  |  | 126 | 0.312 | 0.5899 | Yes |
| 23 | H3F3A |  |  | 132 | 0.304 | 0.6059 | Yes |
| 24 | ASCL1 |  |  | 145 | 0.290 | 0.6083 | Yes |
| 25 | CCND2 |  |  | 149 | 0.288 | 0.6264 | Yes |
| 26 | RNASEH2B |  |  | 158 | 0.282 | 0.6353 | Yes |
| 27 | CHD7 |  |  | 166 | 0.277 | 0.6455 | Yes |
| 28 | HELLS |  |  | 171 | 0.271 | 0.6605 | Yes |
| 29 | BAZ2B |  |  | 190 | 0.259 | 0.6498 | No |
| 30 | HSPA5 |  |  | 447 | -0.350 | 0.2260 | No |
| 31 | NES |  |  | 509 | -0.435 | 0.1537 | No |
Table: GSEA details [plain text format]

  

Fig 2: RICHARDS\_NOWAKOWSKI\_SCIENCE\_2017\_MGE\_IPC3\_UPREG: Random ES distribution      
 Gene set null distribution of ES for **RICHARDS\_NOWAKOWSKI\_SCIENCE\_2017\_MGE\_IPC3\_UPREG**
